# Light-patterning of synthetic tissues with single droplet resolution

**DOI:** 10.1038/s41598-017-09394-9

**Published:** 2017-08-24

**Authors:** Michael J. Booth, Vanessa Restrepo Schild, Stuart J. Box, Hagan Bayley

**Affiliations:** Chemistry Research Laboratory, University of Oxford, Oxford, OX1 3TA UK

## Abstract

Synthetic tissues can be generated by forming networks of aqueous droplets in lipid-containing oil. Each droplet contains a cell-free expression system and is connected to its neighbor through a lipid bilayer. In the present work, we have demonstrated precise external control of such networks by activating protein expression within single droplets, by using light-activated DNA to encode either a fluorescent or a pore-forming protein. By controlling the extent of activation, synthetic tissues were generated with graded levels of protein expression in patterns of single droplets. Further, we have demonstrated reversible activation within individual compartments in synthetic tissues by turning a fluorescent protein on-and-off. This is the first example of the high-resolution patterning of droplet networks, following their formation. Single-droplet control will be essential to power subsets of compartments within synthetic tissues or to stimulate subsets of cells when synthetic tissues are interfaced with living tissues.

## Introduction

Droplet networks are soft materials consisting of multiple aqueous droplets bound to each other by lipid bilayers, called droplet interface bilayers (DIBs)^1, 2^. DIBs are formed from aqueous droplets submerged in a lipid-containing oil. The droplets acquire a lipid monolayer, and when brought together form a bilayer. Soft biodevices have been generated from droplets networks, including batteries^3^, electronic devices^4^, bioreactors^5^, logic gates^6^ and tissue-mimics^7, 8^. Droplet networks can also be generated in a bulk aqueous solution^9–11^. The fragility of these droplet bilayer systems is of concern, but has been addressed by using organogels^12^, hydrogel shells^13, 14^ and PEGylated lipids^8^. Droplet networks have potential applications in biotechnology, for instance in drug delivery or in tissue repair^15^. Droplet networks will have advantages over single compartment devices, such as vesicles, through the use of binary or higher order compartmentalization and the ability to build multiple functions into one device. Drug delivery using simple aggregates of aqueous droplets has already been approved for clinical use^16^.

Several techniques have been devised for generating droplet networks. Networks of nL-sized droplets can be created manually with pipettes or syringes^3, 17^. However, this is slow and laborious. Optical tweezers^18^ and magnets, when the droplets contain magnetic beads^19^, can also be used to accurately place droplets, but this is difficult to automate for the production of large networks. Microfluidics have also been used to form droplet networks^20–22^. This allows the high-throughput formation of large networks, but it is challenging to accurately pattern the droplets. We previously developed a droplet printer, which can create large patterned droplet networks in an automated manner from pL-sized droplets^7^. Aqueous droplets are ejected from glass capillary nozzles into a lipid-containing oil. Two types of droplet, dispensed from two different nozzles, can be patterned into defined locations within the network. However, with this technique, it is difficult to position droplets within a network at single-droplet resolution. Additionally, a more sophisticated multi-nozzle printer would be needed to pattern more than two types of droplets in a single network. With all of these formation methods, after droplet networks have been formed the initial patterning cannot be altered, without replacing droplets in the network. With large droplet networks and those with very small droplets, the replacement of droplets is very difficult.

We recently developed a tightly regulated light-activated DNA (LA-DNA) system. When used with an *in vitro* transcription/translation (IVTT) system, LA-DNA leads to protein expression after UV irradiation^8^. Light-activated transcription and translation has been previously demonstrated^23, 24^. However, these methods either don’t show tight regulation or are not compatible with lipid bilayer systems. By incorporating LA-DNA and an IVTT system into aqueous droplets, we formed printed droplet networks that expressed proteins in defined regions within the networks. We called these networks ‘synthetic tissues’, and this was the first example of control of the expression of protein within a droplet network with an external signal^8^. Light-patterning of microfluidically generated droplet arrays has been previously demonstrated^25^, however no bilayers were present and the resolution was low. Here, we have utilized the LA-DNA system to pattern synthetic tissues at single-droplet resolution, after their formation. Further, by activating single droplets in the same network to different extents, we generated networks that possess four different levels of expressed protein within the droplets, which would not be possible to generate directly with the current two-nozzle droplet printer. Additionally, we incorporated a photoswitchable fluorescent protein^26^ into the synthetic tissues and demonstrated reversible patterning. The control of droplet networks with single-droplet resolution, after their formation, is an important step towards their development as remotely controlled synthetic tissues.

## Results

### Light-activation of individual nL-sized droplets within droplet networks

**Figure 1:**
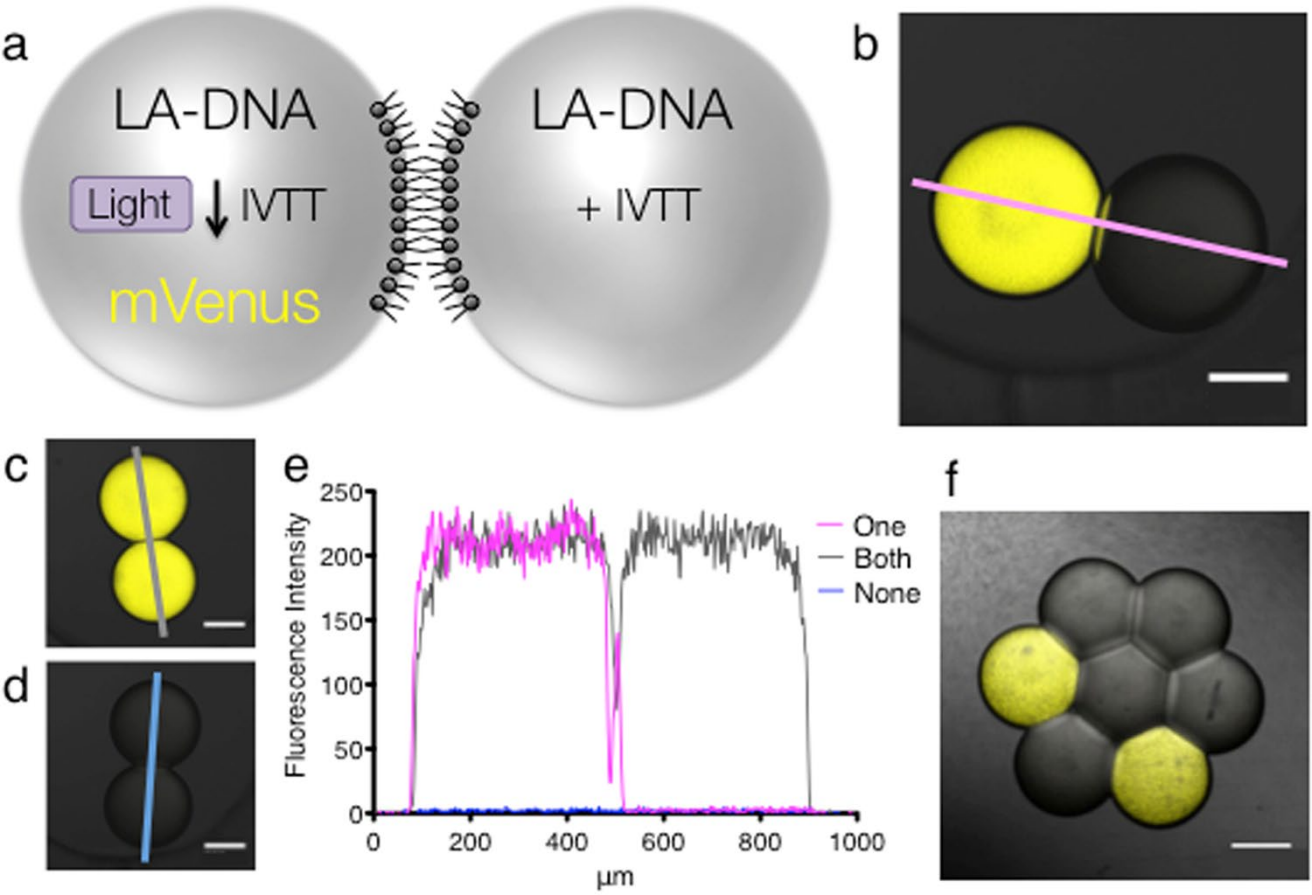
Activation of protein expression in single nL-sized droplets. (**a**) Schematic representing the light-activated expression of the fluorescent protein mVenus in a single droplet of a droplet pair. (**b**) Image of single-droplet activation within a droplet pair. (**c**) Activation of both droplets in a droplet pair. (**d**) Activation of neither of the droplets in a droplet pair. (**e**) Fluorescence intensity line profiles from ‘b’ (pink line), ‘c’ (grey line) and ‘d’ (blue line, on baseline). Scale bars for ‘b–d’, 200 μm (**f**) Activation of only two droplets in a seven-droplet network. Scale bar, 250 μm.

Protein expression can be activated within aqueous droplets, containing LA-DNA and an IVTT system, in an lipid-containing oil by using 365 nm ultraviolet (UV) light^8^. Activation of protein expression within droplets can be initiated with either a 365 nm LED or a fluorescence light microscope with a DAPI (325–375 nm excitation) filter cube (Supplementary Fig. 1). When droplets are incubated in lipid-containing oil, a lipid monolayer forms on the surface. When two such droplets are connected a DIB is formed. Further, when multiple droplets are brought together they form a droplet network, in which each droplet is connected to its neighbor through a DIB. We aimed to activate individual droplets within droplet networks. To do this we used a circular field diaphragm built into a fluorescence light microscope. Using a pair of nL-sized droplets that both contained the yellow fluorescent protein mVenus, we restricted the diaphragm and increased the magnification of the microscope to illuminate different regions of the droplets. We could decrease the diameter of the transmitted light beam to 50 μm, and thereby illuminate both droplets, one droplet, or even a section of a droplet (Supplementary Fig. 2). By adjusting the microscope diaphragm we could set the light beam to different diameters. As we were able to image the fluorescence of only a single droplet, when both droplets contained the same protein, this indicated that we might be able to activate only a single droplet, when both droplets had the potential to be activated. As an initial demonstration of single droplet activation, we prepared nL-droplet pairs in which both droplets contained the IVTT system and LA-DNA encoding the yellow fluorescent protein mVenus. With the diaphragm set to 200 μm, we focused the microscope on a single droplet of the pair. In this manner, only the droplet in the illumination path would be activated by the UV light from the DAPI filter cube. Following activation, we found mVenus expression only in the target droplet (Fig. 1a,b). In addition, we could activate both droplets separately (Fig. 1c) or neither droplet (Fig. 1d). A fluorescence intensity line measurement (Fig. 1e) showed a sharp transition at the location of the bilayer after illumination of a single droplet, demonstrating that expression was tightly restricted to only that droplet. The non-illuminated droplet showed similar background fluorescence levels to those seen when neither droplet was illuminated. To test activation within nL-sized droplet networks, we also manually prepared a hexagonal 7-droplet network, in which all the droplets contained the IVTT system and mVenus LA-DNA. With the microscope diaphragm again set to 200 μm, we created an asymmetric pattern by activating expression in only two of the seven droplets (Fig. 1f). All seven droplets contained the same components, and the pattern was created in the network after its formation. These experiments demonstrated tight light-regulated restriction of expression to a single target droplet.

**Figure 2:**
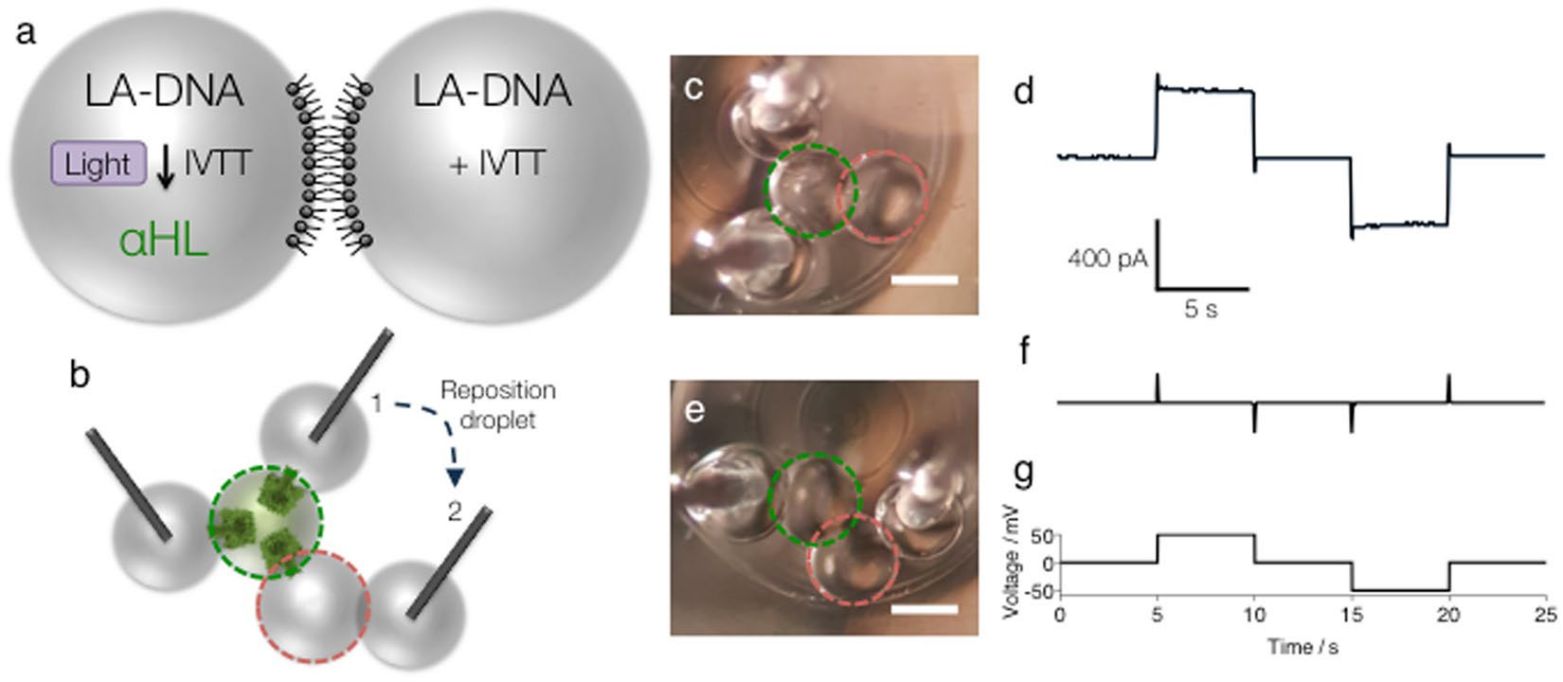
Single-droplet activation in a droplet pair demonstrated by electrical recording. (**a**) Schematic representing the light-activated expression of the pore-forming protein αHL in a single droplet of a droplet pair. (**b**) Schematic showing the use of two electrode-containing droplets to interrogate the droplets in the pair. In position 1, ions can pass through the ‘ON’ droplet of the pair (droplet encircled in green). When one of the electrode-containing droplets is moved to position 2, the ion flux can no longer move through the system, as the ‘OFF’ droplet (droplet encircled in red) of the pair does not contain αHL. (**c**) Image of a droplet network with electrode droplets in position 1. (**d**) Electrical recording performed in position 1. (**e**) Image of a droplet network with electrode droplets in position 2. (**f**) Electrical recording performed in position 2. (**g**) Voltage protocol used for ‘d’ and ‘f’. Scale bars in ‘c’ and ‘e’, 200 μm.

We next sought to test single-droplet activation with a different type of protein and an alternative measurement technique. We expressed the transmembrane protein pore α-hemolysin (αHL), which can spontaneously heptamerise in a lipid bilayer and form a 1.4 nm (diameter) pore^27^. With electrodes in the aqueous droplets, in lipid-containing oil, a voltage can be applied to a droplet pair and the resulting ionic current arising from inserted protein pores measured. We used electrical recording to demonstrate the expression of the αHL protein in a single droplet of a droplet pair (Fig. 2). A droplet pair was prepared from two nL-sized droplets, both of which contained the IVTT system and LA-DNA encoding αHL. One droplet of the pair (the ON droplet) was irradiated with the microscope diaphragm set to 200 μm, which triggered protein expression. To interrogate the droplets in the pair we used two electrode-containing (EC) droplets (Fig. 2b). First, two separate bilayers were formed between the ON droplet and two EC droplets (Fig. 2c,d). When a voltage was applied across these three droplets, a large current was measured. This indicated that the αHL protein had been expressed in the ON droplet and had inserted into the two new bilayers formed with the EC droplets. To test for expression in the OFF droplet, one of the EC DIBs was removed and a new bilayer was formed with the OFF droplet (Fig. 2e,f). When a voltage was applied across the four droplets, no current was measured. This demonstrated that no protein had inserted into the new bilayer, which meant that no αHL had been expressed in the OFF droplet. From these electrical recordings (Fig. 2c–g), we concluded that the activation of expression, heptamerization and membrane insertion of αHL occurred only in the irradiated (ON) droplet. Single-droplet activation of the expression of a membrane protein demonstrates that the method is not restricted to fluorescent proteins and should be widely applicable.

### Light-activation of individual pL-sized droplets within synthetic tissues

**Figure 3:**
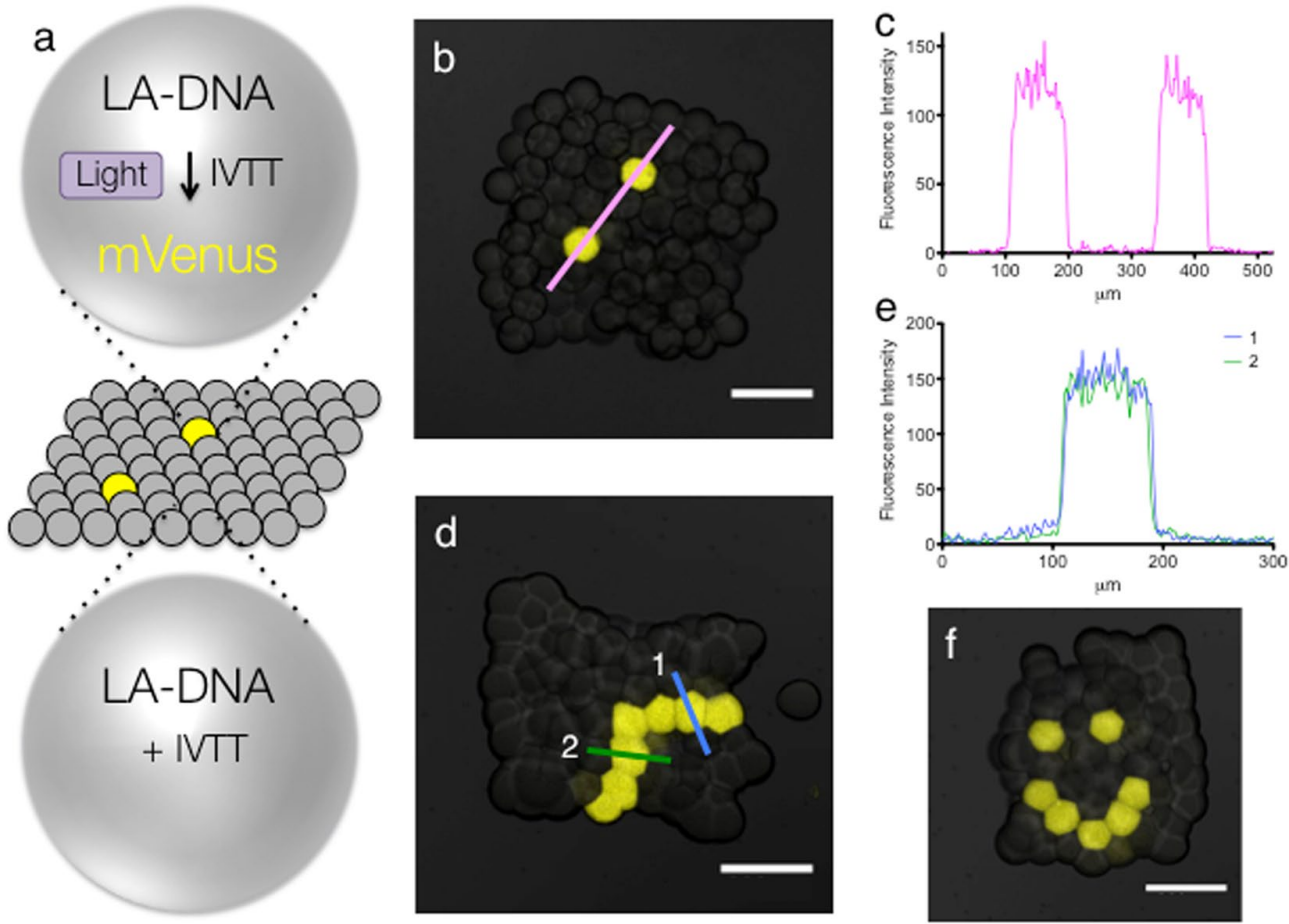
Light-patterned protein expression in single pL-sized droplets within synthetic tissues. (**a**) Schematic representing the light-activated expression of the fluorescent mVenus protein in single droplets of a synthetic tissue. (**b**) Light-activated expression of mVenus in two separate droplets of a synthetic tissue. (**c**) Fluorescence intensity line profile through the two activated droplets in ‘b’. (**d**) Light-activated expression of a single-droplet-wide pathway in a synthetic tissue. (**e**) Fluorescence intensity line profiles through two single droplets of the pathway in ‘d’, showing that the width of the fluorescent pathway is around 100 μm, which is the width of a single droplet. (**f**) Light-activated expression of a face-like pattern in a synthetic tissue. Scale bars, 200 μm.

We previously developed a droplet printer, that can create droplet networks composed of pL-sized droplets^7^. When the networks possess a minimal cellular function, such as the ability to express proteins, we call them synthetic tissues. Using two different printing nozzles, patterned networks can be generated. However, patterning at single-droplet resolution with two nozzles is difficult. By activating individual droplets in printed synthetic tissues after they have been formed, it would be possible to create high-resolution patterns not attained before. To do this we again used the circular field diaphragm. Using a droplet network of pL-sized droplets that contained the yellow fluorescent protein mVenus, we restricted the diaphragm and increased the magnification of the microscope to illuminate different regions of the network. We were able to decrease the diameter of the transmitted light beam to 50 μm, and thereby illuminate only a single droplet of ~75 μm﻿ diameter (Supplementary Fig. 3). As we were able to image the fluorescence of only a single droplet, when all the droplets contained the same protein, this indicated that we might be able to activate only a single droplet, when they all had the potential to be activated. Initially, we printed a synthetic tissue in which all the pL-sized droplets contained LA-DNA encoding mVenus. We printed two layers of droplets to pathway, demonstrated single-droplet resolution. The focus of all images is on this bottom layer of droplets. We irradiated two non-adjacent droplets within this synthetic tissue, with the microscope diaphragm set to 50 μm (Fig. 3a). A fluorescence intensity measurement along a line through the synthetic tissue demonstrated that expression of mVenus was restricted to only these two droplets (Fig. 3b,c). Background fluorescence was observed in the neighboring droplets.

To demonstrate the accuracy of the technique, we again printed synthetic tissues, in which all of the pL-sized droplets contained LA-DNA encoding mVenus. By setting the microscope diaphragm to 50 μm and illuminating several droplets, one at a time, patterns of fluorescence could be generated through the activation of protein expression (Fig. 3d–f). A pathway one droplet wide from one side of the network to an adjacent side could be formed (Fig. 3d,e). Fluorescence intensity measurements of lines across the pathway, demonstrated single-droplet resolution. A few neighboring droplets showed a slight increase in fluorescence over the background (Fig. 3e), due to difficulties in directing the beam at atypically shaped droplets. However, there was a >10-fold contrast in expression between these droplets and those in the single-droplet pathway. Additionally, a face-like pattern was activated in a synthetic tissue (Fig. 3f), which demonstrates a more technically challenging pattern that would be extremely difficult to form by printing alone.

### Light-patterning of different levels of protein expression in single pL-sized droplets within synthetic tissues

Because our current droplet printer only has two nozzles, it is only possible to pattern two different types of droplet within the same network. To print more than two types of droplet, a more sophisticated multi-nozzle printer would be required. We next aimed to generate networks that contained multiple types of droplets (three or more), which is not possible with the present version of the printer. In IVTT reactions, a reduction in the concentration of template DNA reduces the yield of expressed protein (see New England Biolabs PURExpress manual^28^). We demonstrated that when LA-DNA is irradiated with UV for less time, less RNA is produced, presumably because the blocking streptavidin is cleaved to a lesser extent (Supplementary Fig. 4). This indicated that we might activate individual droplets within synthetic tissues for different amounts of time to produce different quantities of protein in each droplet. This would allow us to pattern synthetic tissues with multiple types of droplet after the formation of the networks.

Initially, we printed synthetic tissues in which all of the pL-sized droplets contained LA-DNA encoding mVenus. We then set the microscope diaphragm to 50 μm and activated single droplets for 10–60 seconds. Following expression we quantified the level of fluorescence within each droplet (Supplementary Fig. 5). The measured fluorescence intensity, which serves as an indicator of the level of protein expression, increases with UV illumination duration.

**Figure 4:**
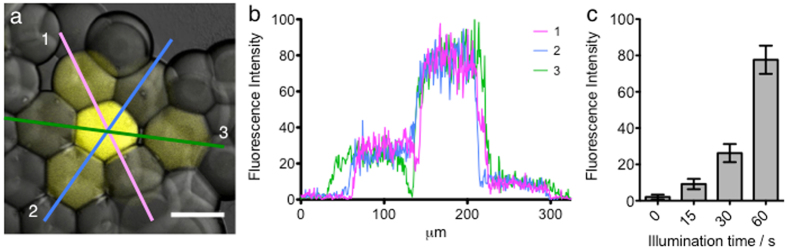
Light-patterning different levels of protein expression in multiple single pL-sized droplets within a synthetic tissue. (**a**) Droplets within a printed synthetic tissue, containing LA-DNA encoding mVenus, were light-activated for different durations, producing different levels of expressed protein in each droplet. The central droplet was activated for 60 s, and the surrounding six droplets were illuminated for 30 s and 15 s, in an alternating pattern. The droplets surrounding the seven activated droplets were not illuminated. Scale bar, 75 μm. (**b**) Three fluorescence intensity line profiles through the hexagon shape in ‘a’. (**c**) Plot showing the mean and standard deviation of the fluorescence intensity across each droplet type, an indicator of the level of expression, for a particular droplet type against the corresponding illumination time.

We next aimed to produce synthetic tissues with patterns of droplets, each with a pre-determined level of protein. We again printed a synthetic tissue in which all of the pL-sized droplets contained LA-DNA encoding mVenus. We set the microscope diaphragm to 50 μm and activated seven droplets in the shape of a hexagon with three different illumination durations, to produce a network of droplets with three different concentrations of protein or no protein (Fig. 4). The middle droplet of the seven was illuminated for 60 s and the six surrounding it were illuminated for 30 s or 15 s, in an alternating pattern (Fig. 4a). The droplets surrounding these seven were not illuminated. We quantified the fluorescence following protein expression under our standard conditions of 18 hours at 25 °C, and observed four different types of droplet (Fig. 4b,c). The highest expression was observed in the droplet illuminated for 60 s and only background fluorescence was observed in the droplets that weren’t illuminated. However, two different levels of expression were observed for the droplets subjected to 15 s or 30 s of illumination (Fig. 4c): respectively, 12 ± 4% and 34 ± 7% of the expression from 60 s of illumination. Four different printer heads would have been required to print this structure without the aid of subsequent light activation of expression.

### Reversible patterning of networks of nL-droplets

The activation of protein expression within droplets is an irreversible process. Ideally, we would like to re-pattern networks at single-droplet resolution, after their formation, and subsequently recover the initial pattern. Therefore, we sought to demonstrate reversible patterning at single-droplet resolution. To this end, we used the reversible fluorescent protein rsTagRFP^26^. rsTagRFP exhibits photoswitchable red fluorescence. The fluorescence can be turned to an OFF state with yellow light (567 nm maximum) and then back to an ON state with blue light (440 nm maximum). This reversible fluorescence has been shown to work for over 15 ‘ON-OFF’ cycles^26^. Newly synthesized rsTagRFP is found in the ON state, but when equilibrated at room temperature only about one-third of rsTagRFP molecules are in the ON state. The OFF and ON states have relaxation half-times to the equilibrated state of 46 and 65 min^26^. We cloned an rsTagRFP gene into our PURExpress template and expressed it in bulk. We optimised a set of filter cubes on our microscope to image the fluorescence and reversibly switch the fluorophore ON and OFF (see Methods). We then repeatedly deactivated and activated the protein activity in nL-sized droplets in lipid-containing oil (Supplementary Fig. 6), which demonstrated that rsTagRFP can be functionally expressed with an IVTT system in bulk, inserted into aqueous droplets and then controlled optically.

Our microscope diaphragm could be reduced to less than the diameter of a single nL-sized droplet (Supplementary Fig. 2). Additionally, the diaphragm could restrict the shape of the laser beam to either a circle or a square. This allowed us to pattern shapes within a single droplet, and watch the diffusion of the fluorescent protein across the whole droplet. Initially, starting with an activated droplet, the diaphragm was set to deactivate the fluorescence within a 200 μm square region of a single nL-sized droplet (Supplementary Fig. 7). We then observed the fluorescent protein from the remaining volume of the droplet diffuse into the bleached volume within 120 seconds. A complementary experiment was also performed. We set the diaphragm to produce a circular spot of 100 μm diameter. We then activated a cylinder of fluorescence within a single deactivated nL-sized droplet, and observed the activated fluorescent protein diffuse throughout the entire droplet within 180 seconds (Supplementary Fig. 8). These results are in line with the diffusion rates reported for GFP^29, 30^.

**Figure 5:**
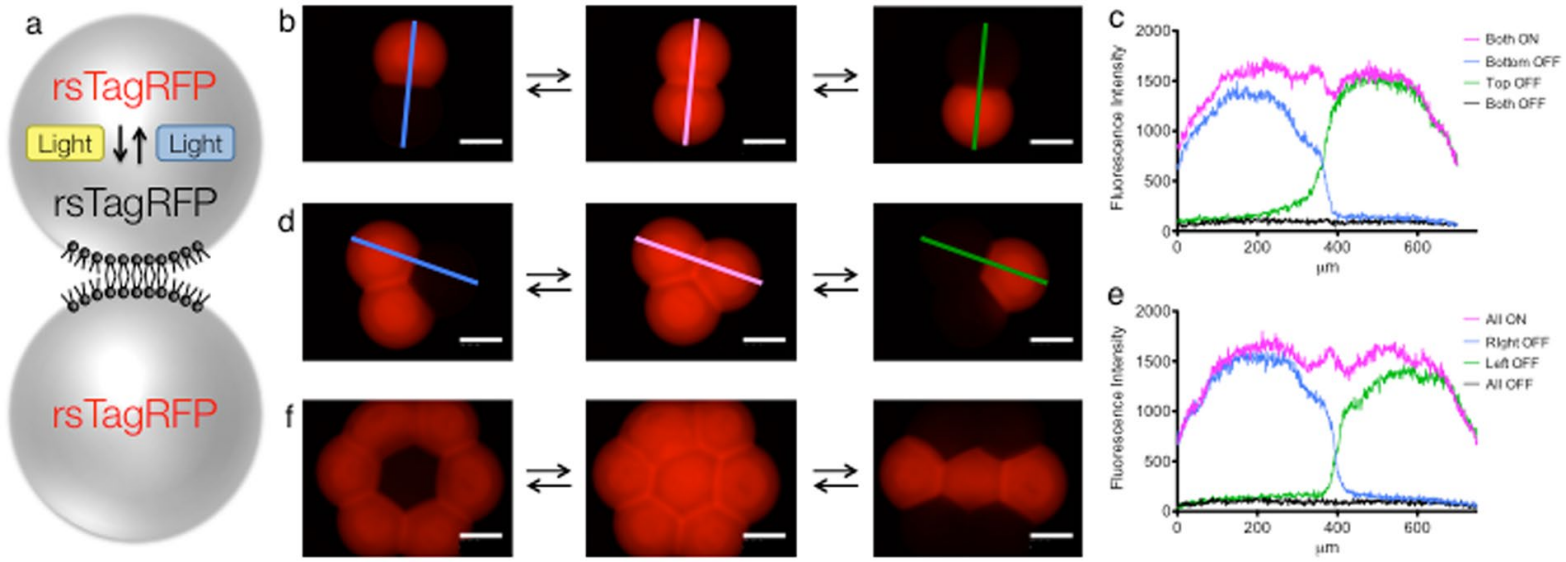
Reversible activation of the fluorescent protein rsTagRFP in single nL-sized droplets within a droplet pair and within droplet networks. (**a**) Schematic representing the activation and deactivation of the reversible fluorescent protein rsTagRFP in a droplet pair. (**b**) The fluorescence of single droplets in a droplet pair can be turned OFF with light, the whole system reactivated, then a different droplet turned OFF. (**c**) Fluorescence intensity line profiles from ‘b’. (**d**) Turning OFF the fluorescence in single droplets of a three-droplet network. (**e**) Fluorescence intensity line profiles from ‘d’. (**f**) Turning OFF the fluorescence in single droplets of a seven-droplet network. Scale bars, 250 μm.

We had now generated a system with which we could reversibly control rsTagRFP’s fluorescence in individual nL-sized droplets with light. We next aimed to reversibly control rsTagRFP at single-droplet resolution within droplet networks consisting of nL-sized droplets. This would allow us to reversibly pattern droplet networks after their formation, a step beyond our previous demonstration of irreversible control of the IVTT reaction in droplet networks. Initially, droplet networks that contained activated rsTagRFP were generated. Reversible control was demonstrated by deactivating single droplets within a network, then reactivating the entire network and then deactivating a different single droplet, or set of single droplets (Fig. 5). We were able to accurately and reversibly pattern the fluorescence of single nL-sized droplets in droplet pairs (Fig. 5b,c), 3-droplet networks (Fig. 5d,e) and 7-droplet networks (Fig. 5f). A tight OFF state was observed in the droplets that were deactivated, with minimal reduction in the fluorescence of the droplets that were left ON (Fig. 5c,e). A >10-fold contrast was observed between the ON and OFF states. Complementary sets of experiments were also performed. We first deactivated the entire network, activated single droplets, deactivated the entire network again, and then reactivated different single droplets or sets of single droplets (Supplementary Fig. 9). This experiment was performed on nL-sized droplets in droplet pairs (Supplementary Fig. 9b,c), 3-droplet networks (Supplementary Fig. 9d,e) and 7-droplet networks (Supplementary Fig. 9f). Fluorescence of the activated droplets was efficiently turned ON, however there was also a small increase in fluorescence measured in the droplets that were not activated (Fig. 9c,e). This might have been from either the brightfield setting used to move and focus the microscope or stray light hitting the neighboring droplets, which only causes a problem in this instance because of the much faster kinetics of activation than deactivation^26^. However, there was still a >6-fold contrast in the ON state over the OFF state.

### Reversible patterning of pL-sized droplets within synthetic tissues

**Figure 6:**
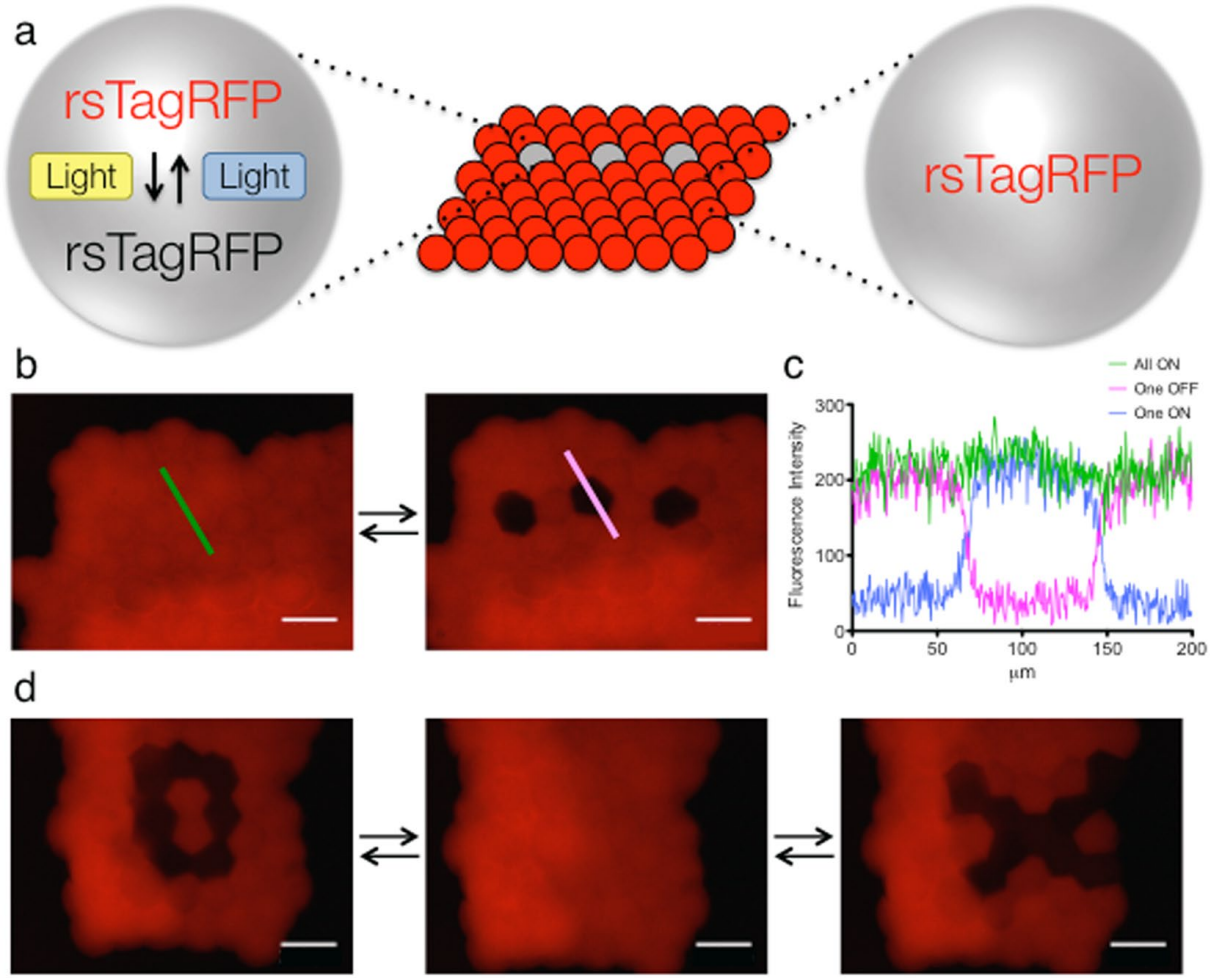
Reversible light-patterning of the fluorescent protein rsTagRFP in single pL-sized droplets within synthetic tissues. (**a**) Schematic representing the deactivation of the reversible fluorescent protein rsTagRFP in a synthetic tissue. (**b**) Deactivation of three separate droplets in a synthetic tissue. (**c**) Fluorescence intensity line profiles of the single droplet deactivation in ‘b’ (green and pink lines) and single droplet activation of the same single droplet from Fig. S8 (blue line). (**d**) Reversible patterning of an ‘O’ and an ‘X’ pattern in pL-droplets in a synthetic tissue. Scale bars, 100 μm.

As previously demonstrated, we could irreversibly pattern the function of single droplets within synthetic tissues (Fig. 3). This allowed us to generate complex droplet networks, after their formation. We next aimed to generate reversible patterns within pL-sized droplets in synthetic tissues. We used the droplet printer to create aqueous droplet networks composed of pL-sized droplets containing the IVTT system and DNA encoding rsTagRFP. We printed two layers of droplets to ensure the bottom layer was fully packed. The focus of all images is on this bottom layer of droplets. rsTagRFP was then expressed within these synthetic tissues, and its fluorescence was measured (Fig. 6). The previously optimized activation and deactivation scheme was used with the microscope diaphragm set to 50 μm. We deactivated three non-adjacent pL-sized droplets within a printed synthetic tissue (Fig. 6a–c). Measurement of fluorescence intensity demonstrated an efficiently generated OFF state displaying background fluorescence in just the three droplets, while the fluorescence of the neighboring droplets was unchanged. To test the ability to turn ON single droplets, we first deactivated the entire synthetic tissue, and then reactivated a single droplet, with the diaphragm again set to 50 μm (Supplementary Fig. 10). The fluorescence intensity generated in the droplet was similar to that found in each droplet when the entire synthetic tissue was activated (Fig. 6c and Supplementary Fig. 10b). Fluorescence intensity measurements from the deactivation and activation of single droplets demonstrated the ability to accurately and reversibly control pL-sized droplets in synthetic tissues. To further demonstrate accuracy and reversibility, we produced more technically challenging patterns. With the diaphragm set to 50 μm, we were able to create, reversibly, ‘O’ and ‘X’ patterns of fluorescence at single droplet resolution (Fig. 6d). This demonstrates that not only can we pattern at single-droplet resolution in droplet networks, but we can reversibly and accurately alter the pattern at will.

## Discussion

Here we have demonstrated, for the first time, that pre-formed droplet networks can be externally controlled and patterned at single pL-droplet resolution. Spatially accurate control can occur at either the level of transcription or after protein formation. Irreversible transcriptional control was demonstrated with LA-DNA by using UV light, while control of protein activity was established with a reversible fluorescent protein by using yellow and blue light. We demonstrated high contrast between activated single droplets and their non-activated neighbors. Further, we have obtained networks with graded levels of expression, generating the first printed droplet network containing more than 2 types of droplets. This high-resolution remote control of droplet networks is an important step forward in their development as biodevices. We have previously incorporated light-activated protein pumps into droplet arrays to generate patterned current generation^31^. In accord with our use of the model light-activated protein rsTagRFP, we might now incorporate these light-activated protein pumps into synthetic tissues to generate power at single droplet resolution. By incorporating other proteins that are light-activatable into droplet networks, we could gain reversible enzymatic^32, 33^, membrane channel^34, 35^ or transcriptional activity^36, 37^. It will also be of interest to activate single droplets in three dimensions, within droplet networks. This might be achieved with the construction of a dual color DNA promoter.

We envisage interfacing synthetic tissues with living tissue. For example, synthetic tissues controlled at single-droplet resolution might be used to stimulate single or multiple cells, to precisely activate neuronal pathways. In this case, rather than measure the fluorescence or electrical conductivity of the synthetic tissues, we would measure changes in the activities of target cells or tissues. In order to reach this goal many challenges will have to be overcome, including the stability of synthetic tissues over longer time scales and the incorporation of additional functional proteins.

## Methods

### DNA preparation

LA-DNA was prepared as previously described^8^. DNA encoding mVenus (Plasmid #27793) and rsTagRFP (Plasmid #31927) was purchased from Addgene. DNA encoding the α-hemolysin-NN mutant^38^ was used for the electrical studies. rsTagRFP was cloned into the PURExpress control template and then amplified by PCR into a linear transcription template as previously described^8^, by using an unmodified T7 promoter sequence as the forward primer. Gel electrophoresis and T7 transcription were performed as previously described^8^.

### Light-activation of droplets

LED activation of protein expression was performed as previously described^8^. To illuminate individual droplets in droplet networks a Leica DMi8 wide-field light microscope was used, with a HCX PL FL L 40x lens and an illumination field diaphragm. To activate protein expression from LA-DNA, illumination was carried out for 1 minute (unless otherwise stated) with a DAPI filter cube, ¼ shutter intensity and 10% illumination intensity. To deactivate rsTagRFP we used a TXR filter cube (15 s) and to activate protein rsTagRFP we used a GFP filter cube (5 s), both with ¼ shutter intensity and 100% illumination intensity. To minimise quenching of the fluorescence upon imaging rsTagRFP-containing droplet networks we used a DSRed filter cube with ¼ shutter intensity and 17% illumination intensity. For most experiments, a circular illumination field setting of 2 was used for nL-sized droplets and a circular field setting of 1 was used for pL-sized droplets. For illuminating a square or circle inside a nL-sized droplet, a field setting of 1 was used with either a square or circular diaphragm. Any brightness or contrast changes made to the rsTagRFP images were the same within any image set.

### Confocal Microscopy

Confocal fluorescence microscopy was used to image the protein expression experiments. The confocal microscope (Leica, SP5) was used as previously described^8^. The settings used for mVenus were: 514 nm laser at 30%, PMT detector between 525–575 nm and smart gain at 800. All fluorescence images were analysed by using Fiji (ImageJ). Any brightness or contrast changes made to the images were the same within any image set.

### Preparation of lipids

A lipid mixture comprising a 10% molar fraction of DPPE-mPEG2000 (Avanti, 16:0 PEG2000 PE) in DPhPC (Avanti, 4ME 16:0 PC) was prepared as previously described^8^. The total concentration of lipid was 1 mM in all experiments. Three different volume ratios of hexadecane and silicone oil were used, 50:50 (hexadecane:silicone oil, all nL-sized droplet experiments), 40:60 (pL-sized droplets in synthetic tissue experiments with rsTagRFP and LA-DNA experiments in Figs 3b and 4) and 30:70 (pL-sized droplets in synthetic tissue experiments with LA-DNA in Fig. 3d,f).

### Electrical recording

Silver wires (100 μm diameter, Sigma) were soldered into male crimp-terminals (RS Components). The tips of the wires were incubated in sodium hypochlorite, NaClO (10% active chlorine, Sigma) for 45 min to form Ag/AgCl electrodes. The tips of the electrodes were coated with a hydrogel layer by pipetting melted agarose (2% low-melt agarose, Sigma) onto their surfaces. Each electrode was plugged into a female crimp-terminal, which was attached to a micromanipulator (Narishige, NMN-21). The other end of the female crimp-terminal (RS Components) was soldered to a cable that terminated with a male crimp, which was connected to the voltage-clamp amplifier (Triton+, Tecella LLC) via an electrode probe holder (Terrapin, Tecella LLC). The current signal was obtained by an episodic acquisition routine (0 mV, 50 mV, 0 mV, −50 mV, 0 mV, in 5 s intervals) with a feedback resistor of 1 GΩ. Data were acquired at 20 kHz with a 5 kHz filter through the ‘TecellaLab v0.90 type 2’ software. The data was in Tecella’s tlc binary file type and analysed and displayed by using custom software written in LabVIEW^31^.

### Protein expression in nL-sized droplets with LA-DNA

Protein expression was performed with the PURExpress *In Vitro* Protein Synthesis kit (NEB, E6800) according to the manufacturer’s protocol with the addition of Murine RNAse Inhibitor (NEB, MB0314). Poly(ethylene glycol) 4,000 (Sigma) was added to the reactions at a final concentration of 1% w/v. LA-DNA was added to a final concentration of 5–10 ng/μL (mVenus DNA) or 0.5–1 ng/μL (αHL DNA), depending on the activity of the batch of PURExpress.

Droplet (50 nL) formation was performed as previously described^8^ in poly(methyl methacrylate) (PMMA) wells by using a 0.5 μL syringe (Hamilton, 7000.5 KH).

Following mVenus LA-DNA activation, the droplets were incubated at 25 °C for 18 h and the fluorescence signal imaged with the confocal microscope under the settings for mVenus (described above).

Following αHL expression in a droplet pair, the droplets were incubated at 37 °C for 20 min. By using micromanipulators (Narishige, NMN-21) and a stereo-microscope (Nikon, SMZ660), two hydrogel-coated Ag/AgCl wire electrodes were placed inside two additional 50 nL droplets (containing PURExpress reaction mix with water instead of DNA). After 2 min incubation to ensure the formation of monolayers, the two electrode-containing droplets were gently brought into their respective positions on the droplet pair, where they spontaneously formed DIBs. To confirm that bilayers had been formed, the capacitance of the system was measured by applying a triangular voltage waveform. Following confirmation of bilayer formation, the electrical recording protocol described above was applied.

### Protein expression in pL-sized droplets with LA-DNA

PURExpress reactions were prepared as described above. Droplet printing with the reaction mix was performed as previously described^8^, but with new custom software written in LabVIEW (National Instruments). This LabVIEW software combined the previously separate processing-based control programs, manual and map-guided, into one program. Maps for these structures consisted of 2 layers of 9 × 11 droplets. Printing networks two layers thick ensured that the bottom layer was fully packed. Following light-activation, the synthetic tissues were incubated at 25 °C for 18 h and the fluorescence signal imaged with the confocal microscope under the settings for mVenus (described above).

### Experiments with rsTagRFP in droplet networks

rsTagRFP was prepared with the PURExpress synthesis kit by using 10 ng/μL DNA. For nL-sized droplets and networks, rsTagRFP was expressed in bulk at 37 °C for 4 h. nL-sized droplet and droplet network formation was performed as previously described^8^ in poly(methyl methacrylate) (PMMA) chips by using a 0.5 μL syringe (Hamilton, 7000.5 KH). For pL-sized droplets and synthetic tissues, expression was performed within the synthetic tissues after they had been printed. Maps for these structures consisted of 2 layers of 9 × 11 droplets. As above, printing two layers ensured that the bottom layer was fully packed. The synthetic tissues were then incubated at 25 °C for 18 h. Activation, deactivation and imaging was performed as described above.

## Electronic supplementary material

Supplementary Information
