# Supplementary material for: Light-patterning of synthetic tissues with single droplet resolution
Source: Sci Rep. 2017 Aug 24;7:9315. doi: 10.1038/s41598-017-09394-9 (PMC5570938; doi:10.1038/s41598-017-09394-9)
Supplement: Supplementary file 1 — Supplementary Information [file 41598_2017_9394_MOESM1_ESM.pdf]

Supplementary Information

Light-patterning of synthetic tissues with single droplet resolution

Michael J. Booth[1,*], Vanessa Restrepo Schild[1], Stuart J. Box[1], and Hagan Bayley[1,*]
[1]Chemistry Research Laboratory, University of Oxford, Oxford, OX1 3TA, UK.
*Corresponding authors. Email: michael.booth@chem.ox.ac.uk or hagan.bayley@chem.ox.ac.uk

Supplementary Figures

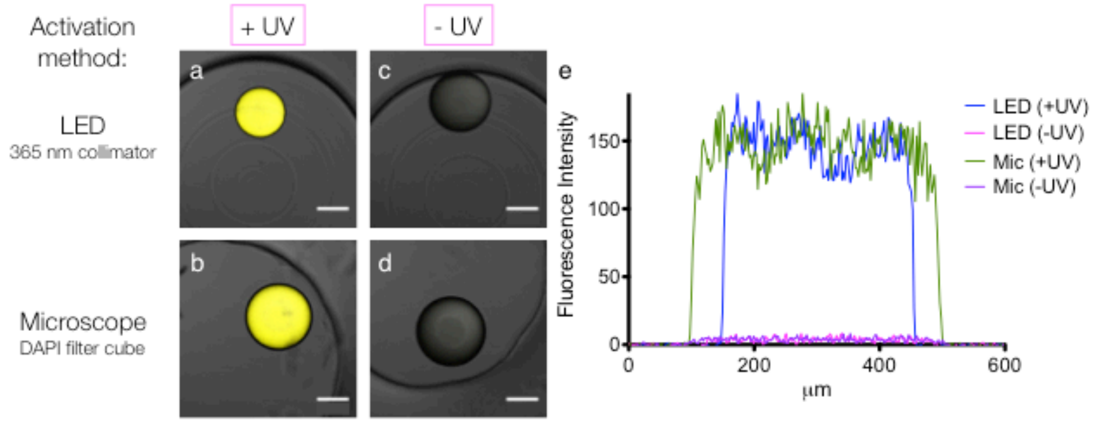

Supplementary Figure 1. Light-activation of protein expression with an LED and a light microscope. Single droplets, containing IVTT mix and LA-DNA encoding mVenus, were activated with either (**a**) a 365 nm LED or (**b**) a DAPI filter cube on a fluorescence microscope. (**c**, **d**) Controls were incubated without UV treatment. Scale bars, 200 µm. (**e**) Fluorescence intensity line profiles demonstrate the activation of protein expression with both methods.

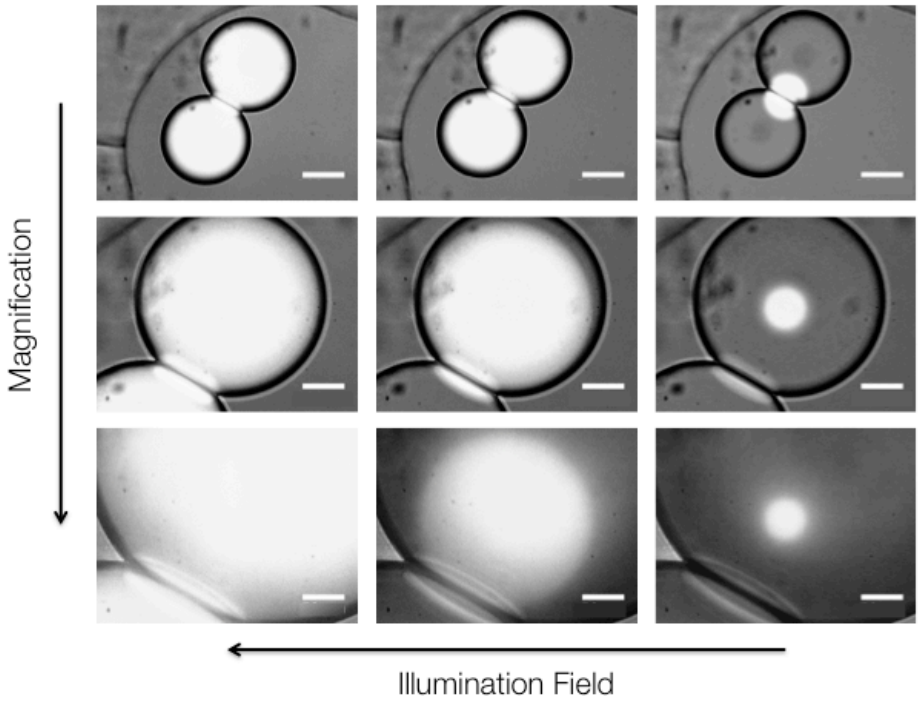

Supplementary Figure 2. Restricted illumination of a droplet pair containing mVenus. By adjusting the magnification and field diaphragm on a fluorescence light microscope, different sections of a droplet pair containing mVenus could be imaged. Here, a GFP filter cube was used to view mVenus. Scale bars, 200 µm (top row), 100 µm (middle row), 50 µm (bottom row).

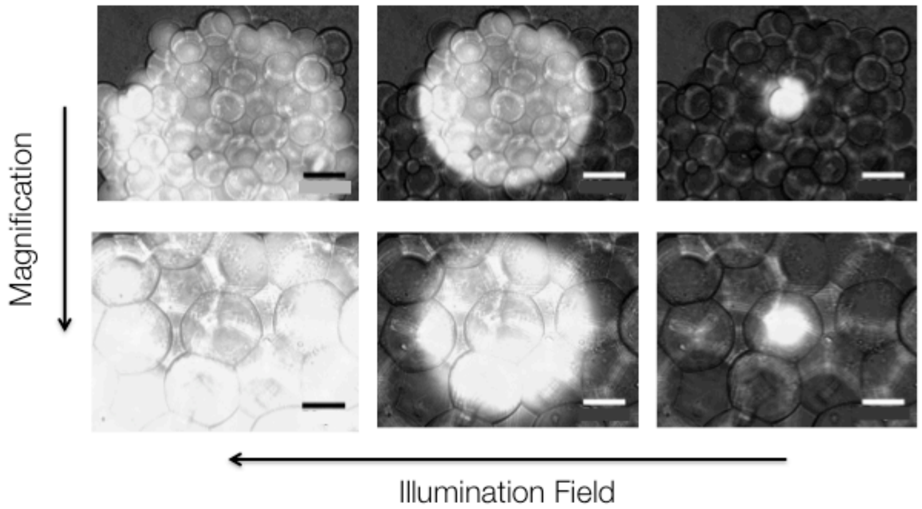

Supplementary Figure 3. Restricted illumination of a droplet network containing mVenus. By adjusting the magnification and field diaphragm on a fluorescence light microscope, different sections of a droplet network containing mVenus could be imaged. Here, a GFP filter cube was used to view mVenus. Figure adapted from Booth *et al.* 2016 *Sci. Adv.* Scale bars, 100 µm (top row), 50 µm (bottom row).

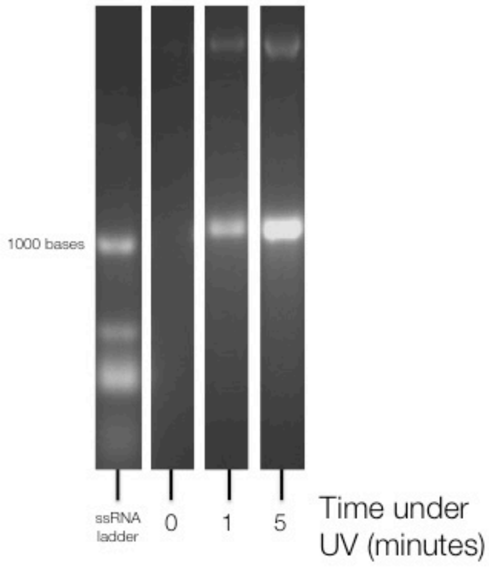

Supplementary Figure 4. RNA yield from different durations of LA-DNA photocleavage. LA-DNA was photocleaved for different amounts of time and then used as a template for T7 RNA transcription. Without UV light, no RNA was produced from LA-DNA. The amount of RNA produced increased with the duration of illumination.

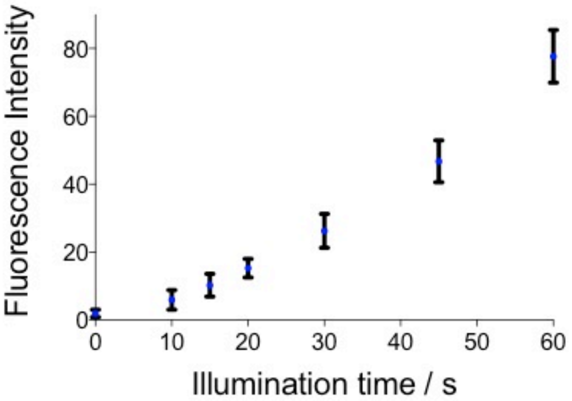

Supplementary Figure 5. Plot showing the dependence of fluorescence intensity (an indicator of mVenus expression) in pL-sized droplets on UV illumination time. pL-sized droplets, containing LA-DNA encoding mVenus, were illuminated with the microscope diaphragm set to 50 µm for 10 to 60 seconds. Following protein expression, the mean and standard deviation of the fluorescence intensity across each droplet was measured.

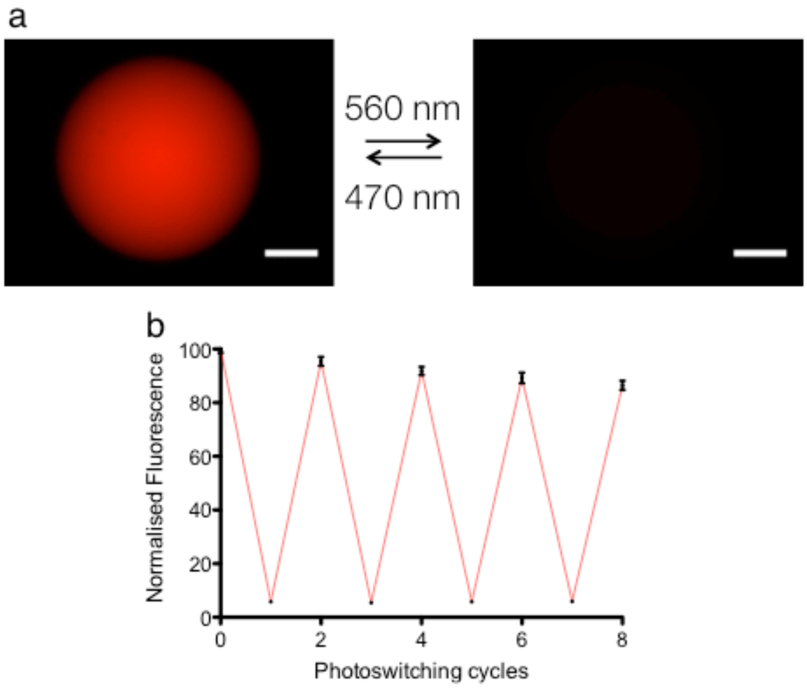

Supplementary Figure 6. Cycling the fluorescence of an aqueous droplet containing rsTagRFP. (**a**) A nL-sized droplet containing the protein rsTagRFP was activated and deactivated with light. Scale bars, 100 µm. (**b**) Fluorescence intensity was measured after each cycle of activation and deactivation and normalized to the first fluorescence measurement. The mean and standard deviation of the fluorescence intensity across the droplet was measured at each cycle.

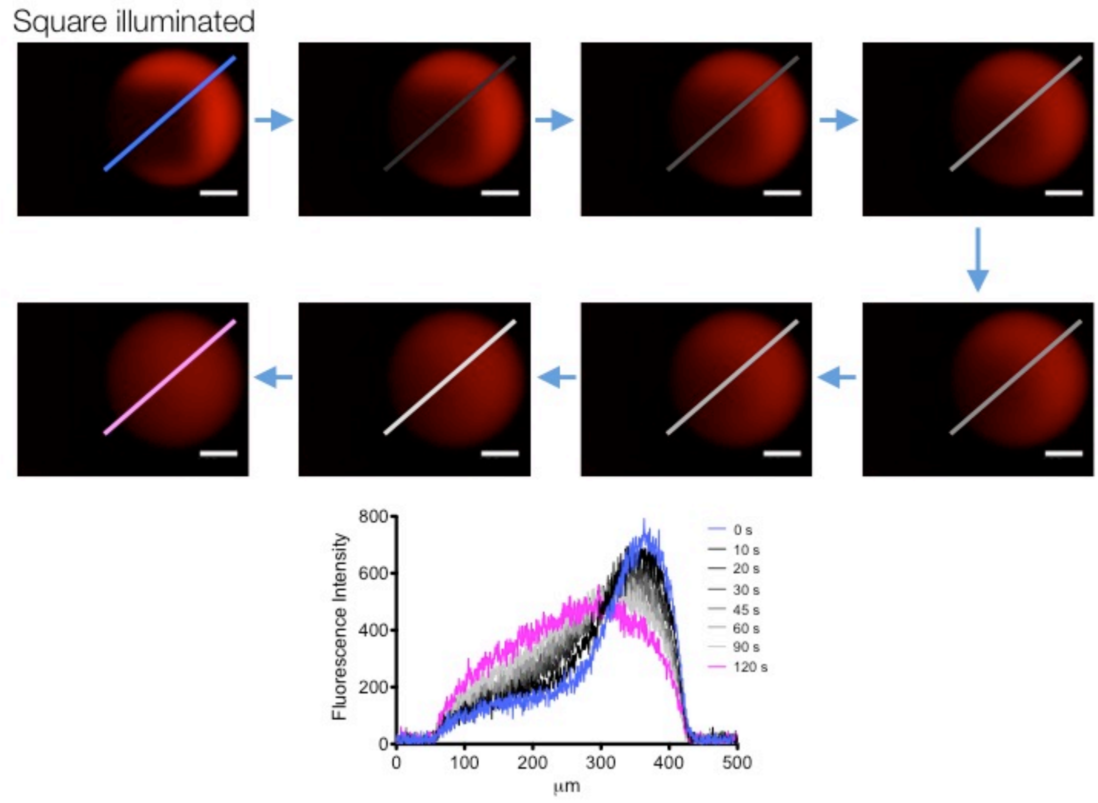

Supplementary Figure 7. Deactivation of rsTagRFP in a rectangular column through a droplet. The microscope diaphragm was set to a square shape to deactivate a rectangular column inside a single droplet. The time series of images covers a period of 2 min, during which the fluorescent protein from the remaining volume of the droplet diffuses into the deactivated section. Fluorescence intensity line profiles quantify the diffusion of the fluorophore across the droplet. Scale bars, 100 µm.

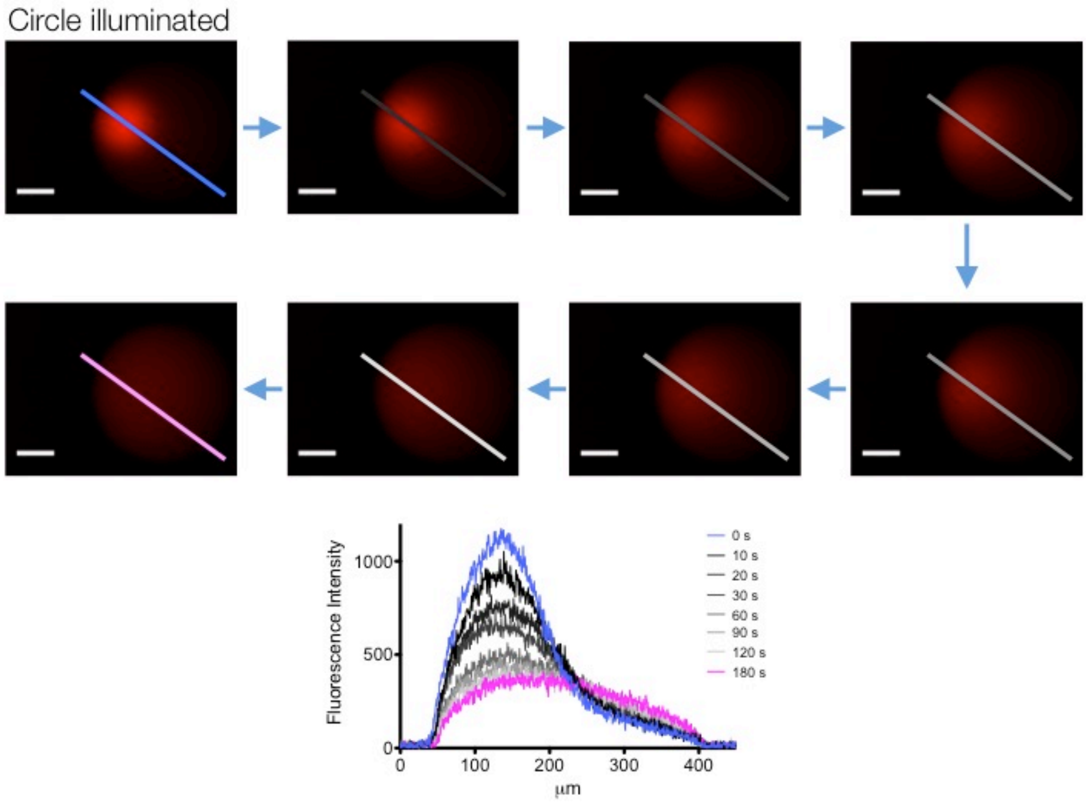

Supplementary Figure 8. Activation of rsTagRFP in a cylindrical section of a droplet. The microscope diaphragm was set to a circular shape to activate a cylindrical column inside a single droplet. The time series of images covers a period of 3 min, during which the activated protein diffuses over the entire droplet. Fluorescence intensity line profiles quantify the diffusion of the fluorophore across the droplet. Scale bars, 100 µm.

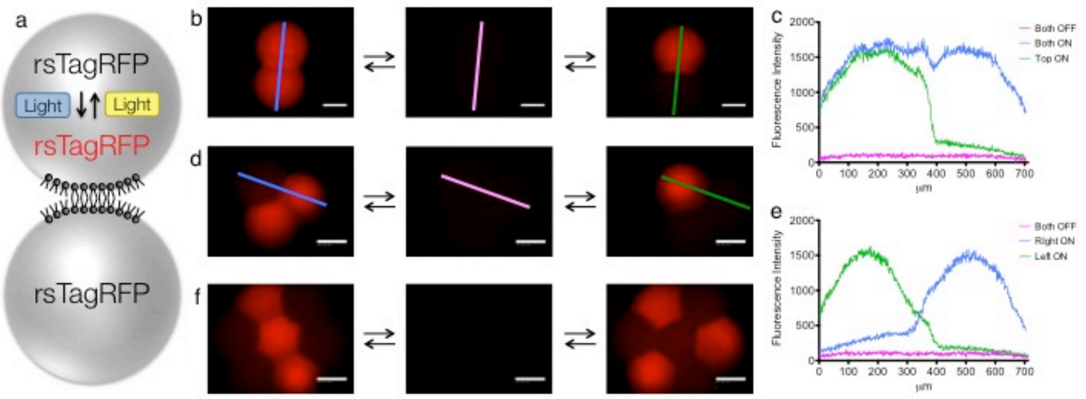

Supplementary Figure 9. Reversible activation of rsTagRFP in single nL-sized droplets in a droplet pair and in small droplet networks. (**a**) Schematic representing the activation and deactivation of the reversible fluorescent protein rsTagRFP in a droplet pair. (**b**) The fluorescence of a single droplet of a droplet pair can be turned ON with light, the whole system deactivated, then a different droplet turned ON. (**c**) Fluorescence intensity line profiles from ‘b’. (**d**) Turning ON the fluorescence in single droplets of a three-droplet network. (**e**) Fluorescence intensity line profiles from ‘d’. (**f**) Turning ON the fluorescence in single droplets of a seven-droplet network. Scale bars, 250 µm.

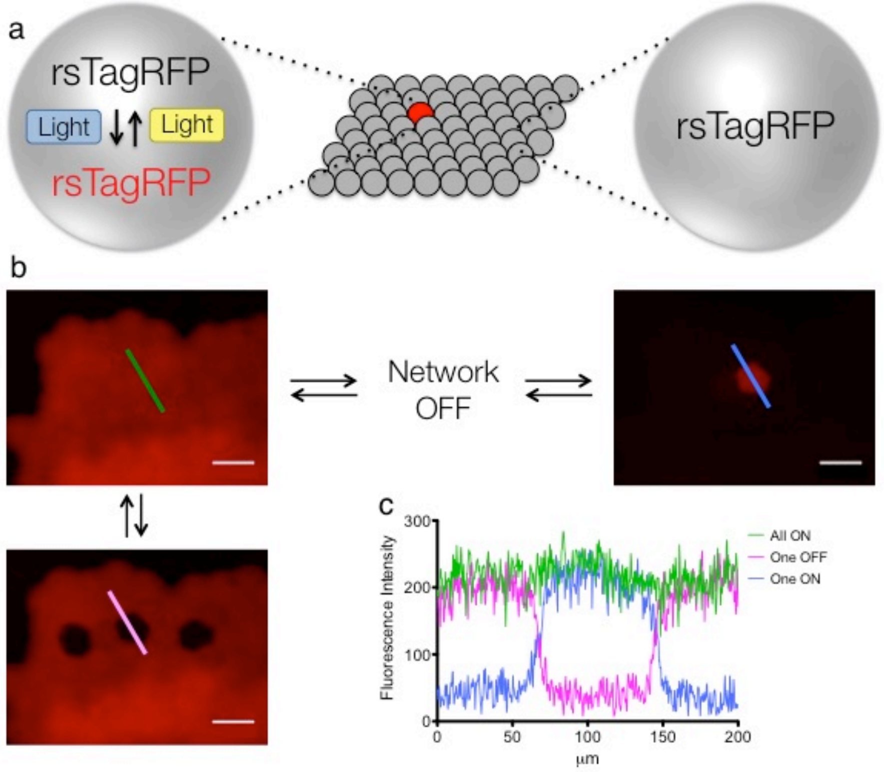

Supplementary Figure 10. Activation of rsTagRFP in a single pL-sized droplet in a synthetic tissue. (**a**) Schematic representing the activation of the reversible fluorescent protein rsTagRFP in a synthetic tissue. (**b**) Deactivation of the fluorescence of an entire synthetic tissue, followed by activation of a single pL-sized droplet. Also shown is the deactivation of three separate droplets in the synthetic tissue. (**c**) Fluorescent intensity line profiles of the fully activated network (green line), single droplet activation (blue line) and single droplet deactivation (pink line). Scale bars, 100 µm.
